# Crystal structure of bis­(μ-2,3,4,5-tetra­fluoro­benzoato-κ^2^ *O*:*O*′)bis­[(1,10-phen­anthroline-κ^2^ *N*:*N*′)(2,3,4,5-tetra­fluoro­benzoato-κ*O*)copper(II)] dihydrate

**DOI:** 10.1107/S1600536814022065

**Published:** 2014-10-11

**Authors:** Junshan Sun

**Affiliations:** a Beijing Key Laboratory for Science and Application of Functional Molecular and Crystalline Materials, Department of Chemistry, University of Science and Technology Beijing, Beijing 100083, People’s Republic of China

**Keywords:** crystal structure, phenanthroline ligands, tetra­fluoro­benzoate ligands, copper(II) complex, hydrogen bonding

## Abstract

In the title compound, [Cu_2_(C_7_HF_4_O_2_)_4_(C_12_H_8_N_2_)_2_]·2H_2_O, the Cu^II^ ion has a square-pyramidal coordination sphere. The basal plane consists of two N atoms [Cu—N = 2.008 (3) and 2.032 (3) Å] from the phenanthroline ligand, and of two carboxyl­ate O atoms [Cu—O = 1.942 (3) and 1.948 (3) Å] from two 2,3,4,5-tetra­fluoro­benzoate anions. Another 2,3,4,5-tetra­fluoro­benzoate anion provides the apical carboxyl­ate O atom [Cu—O = 2.262 (3) Å] and bridges two Cu^II^ ions into a binuclear centrosymmetric dimer. Intra­molecular π–π inter­actions between one of the tetra­fluoro­benzene rings and the middle of the phenenanthroline rings [3.617 (3) Å] stabilize the mol­ecular configuration. O—H⋯O hydrogen bonds between the lattice water mol­ecules and the unbound carboxyl­ate O atoms of the metal complexes leads to the formation of a chain structure parallel to [100].

## Related literature   

For metal complexes with phenanthroline ligands and their derivatives, see: Liu *et al.* (2006 ▶); Kaizer *et al.* (2006 ▶).
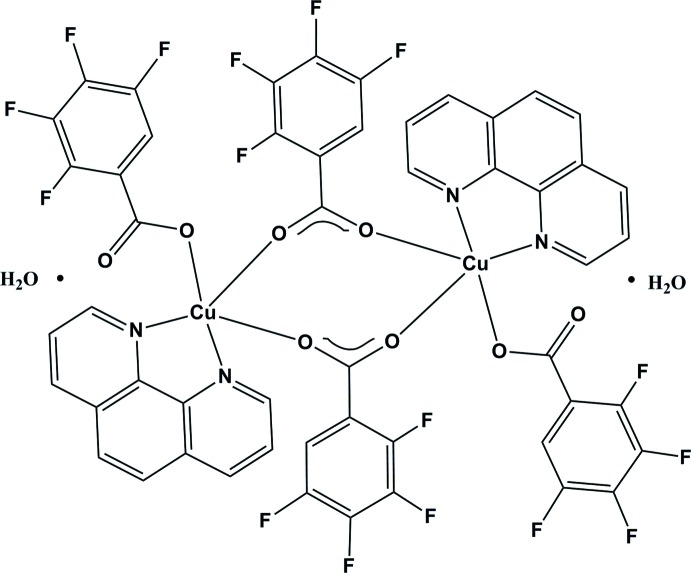


## Experimental   

### Crystal data  

[Cu_2_(C_7_HF_4_O_2_)_4_(C_12_H_8_N_2_)_2_]·2H_2_O
*M* *_r_* = 1295.84
Monoclinic, 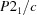

*a* = 7.1880 (8) Å
*b* = 22.611 (2) Å
*c* = 15.2343 (15) Å
β = 103.446 (2)°
*V* = 2408.1 (4) Å^3^
*Z* = 2
Mo *K*α radiation
μ = 1.01 mm^−1^
*T* = 298 K
0.34 × 0.29 × 0.26 mm

### Data collection  

Bruker SMART CCD diffractometer
Absorption correction: multi-scan (*SADABS*; Bruker, 2005 ▶) *T*_min_ = 0.725, *T*_max_ = 0.779
12157 measured reflections
4246 independent reflections
2683 reflections with *I* > 2σ(*I*)
*R*_int_ = 0.049

### Refinement  

*R*[*F* ^2^ > 2σ(*F* ^2^)] = 0.044
*wR*(*F* ^2^) = 0.116
*S* = 1.01
4246 reflections
379 parameters
H-atom parameters constrained
Δρ_max_ = 0.42 e Å^−3^
Δρ_min_ = −0.48 e Å^−3^

### 

Data collection: *SMART* (Bruker, 2005 ▶); cell refinement: *SAINT* (Bruker, 2005 ▶); data reduction: *SAINT*; program(s) used to solve structure: *SHELXS97* (Sheldrick, 2008 ▶); program(s) used to refine structure: *SHELXL97* (Sheldrick, 2008 ▶); molecular graphics: *SHELXTL* (Sheldrick, 2008 ▶); software used to prepare material for publication: *publCIF* (Westrip, 2010 ▶).

## Supplementary Material

Crystal structure: contains datablock(s) I, global. DOI: 10.1107/S1600536814022065/wm5062sup1.cif

**Fig. 1.**
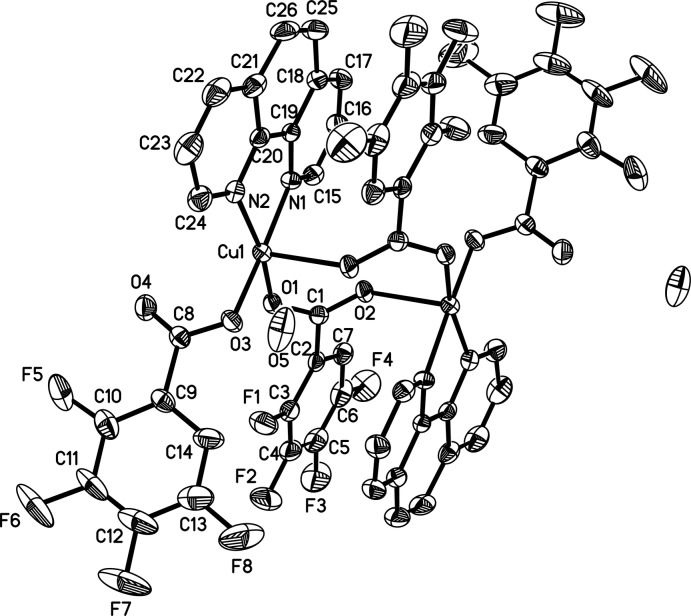
The molecular structure of the title compound, with displacement ellipsoids drawn at the 30% probability level for non-H atoms. The non-labelled atoms are generated by symmetry code –x + 1, –y + 1, –z + 2.

Structure factors: contains datablock(s) I. DOI: 10.1107/S1600536814022065/wm5062Isup2.hkl

x y z . DOI: 10.1107/S1600536814022065/wm5062fig1.tif
The mol­ecular structure of the title compound, with displacement ellipsoids drawn at the 30% probability level for non-H atoms. The non-labelled atoms are generated by symmetry code –*x* + 1, –*y* + 1, –*z* + 2.

**Fig. 2.**
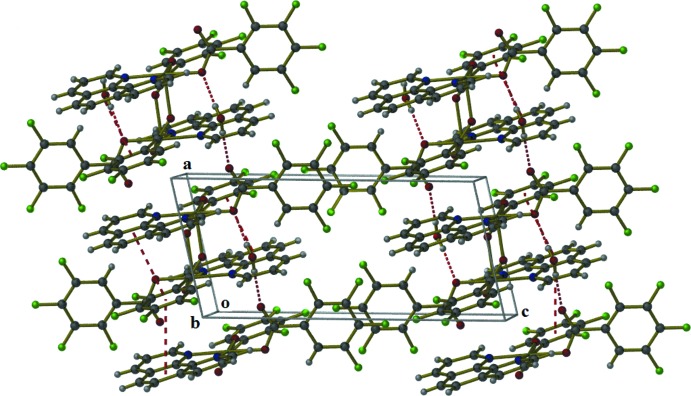
The packing of the molecular entities of the title compound. O—H···O hydrogen-bonding interactions are indicated by dashed lines.

. DOI: 10.1107/S1600536814022065/wm5062fig2.tif
The packing of the mol­ecular entities of the title compound. O—H⋯O hydrogen-bonding inter­actions are indicated by dashed lines.

CCDC reference: 1027857

Additional supporting information:  crystallographic information; 3D view; checkCIF report

## Figures and Tables

**Table 1:** Hydrogen-bond geometry (, )

*D*H*A*	*D*H	H*A*	*D* *A*	*D*H*A*
O5H5*A*O3	0.85	2.08	2.918(5)	168
O5H5*B*O4^i^	0.85	1.95	2.785(5)	168

Symmetry code: (i) 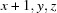.

## supplementary crystallographic information

### S1. Synthesis and crystallisation

The reaction was carried out under solvothermal conditions. 2,3,4,5-tetra­fluoro­benzoic acid (0.388 g, 1 mmol), cupric acetate (0.199 g, 1 mmol) and phenanthroline (0.180 g, 2 mmol) were added into an air-tight vessel together with ethanol and water in a volume ratio of 1:2. The vessel was heated at 393 K for three days and was then cooled down to room temperature with a rate of 10 Kh^-^. The resulting blue solution was filtered and the filtrate was left for sevaral days giving blue block-shaped crystals. Yield: 81%. Elemental analysis (performed with a Perkin Elmer Model 2400 Series II): calc. for C_26_H_12_CuF_8_N_2_O_5_: C 48.26, H 1.61, N 4.40; found: C 48.20, H 1.87, N 4.32.

### S2. Refinement

H atoms of the phenanthroline ring and the anion were placed geometrically (C—H = 0.93 Å) and refined with *U*_iso_(H) = 1.2*U*_eq_(C). H atoms of the water molecule were found from a Fourier difference map and refined with a fixed O—H distance of 0.85 Å and with *U*_iso_(H) = 1.5*U*_eq_(O).

### Crystal data

**Table:**

[Cu_2_(C_7_HF_4_O_2_)_4_(C_12_H_8_N_2_)_2_]·2H_2_O	*F*(000) = 1292
*M**_r_* = 1295.84	*D*_x_ = 1.787 Mg m^−^^3^
Monoclinic, *P*2_1_/*c*	Mo *K*α radiation, λ = 0.71073 Å
Hall symbol: -P 2ybc	Cell parameters from 2862 reflections
*a* = 7.1880 (8) Å	θ = 2.3–25.3°
*b* = 22.611 (2) Å	µ = 1.01 mm^−^^1^
*c* = 15.2343 (15) Å	*T* = 298 K
β = 103.446 (2)°	Block, blue
*V* = 2408.1 (4) Å^3^	0.34 × 0.29 × 0.26 mm
*Z* = 2	

### Data collection

**Table:**

Bruker SMART CCD diffractometer	4246 independent reflections
Radiation source: fine-focus sealed tube	2683 reflections with *I* > 2σ(*I*)
Graphite monochromator	*R*_int_ = 0.049
ω scans	θ_max_ = 25.0°, θ_min_ = 1.6°
Absorption correction: multi-scan (*SADABS*; Bruker, 2005)	*h* = −7→8
*T*_min_ = 0.725, *T*_max_ = 0.779	*k* = −25→26
12157 measured reflections	*l* = −18→16

### Refinement

**Table:**

Refinement on *F*^2^	Primary atom site location: structure-invariant direct methods
Least-squares matrix: full	Secondary atom site location: difference Fourier map
*R*[*F*^2^ > 2σ(*F*^2^)] = 0.044	Hydrogen site location: inferred from neighbouring sites
*wR*(*F*^2^) = 0.116	H-atom parameters constrained
*S* = 1.01	*w* = 1/[σ^2^(*F*_o_^2^) + (0.043*P*)^2^ + 2.2045*P*] where *P* = (*F*_o_^2^ + 2*F*_c_^2^)/3
4246 reflections	(Δ/σ)_max_ = 0.001
379 parameters	Δρ_max_ = 0.42 e Å^−^^3^
0 restraints	Δρ_min_ = −0.48 e Å^−^^3^

### Special details

**Table:**

Geometry. All e.s.d.'s (except the e.s.d. in the dihedral angle between two l.s. planes) are estimated using the full covariance matrix. The cell e.s.d.'s are taken into account individually in the estimation of e.s.d.'s in distances, angles and torsion angles; correlations between e.s.d.'s in cell parameters are only used when they are defined by crystal symmetry. An approximate (isotropic) treatment of cell e.s.d.'s is used for estimating e.s.d.'s involving l.s. planes.
Refinement. Refinement of *F*^2^ against ALL reflections. The weighted *R*-factor *wR* and goodness of fit *S* are based on *F*^2^, conventional *R*-factors *R* are based on *F*, with *F* set to zero for negative *F*^2^. The threshold expression of *F*^2^ > σ(*F*^2^) is used only for calculating *R*-factors(gt) *etc*. and is not relevant to the choice of reflections for refinement. *R*-factors based on *F*^2^ are statistically about twice as large as those based on *F*, and *R*- factors based on ALL data will be even larger.

### Fractional atomic coordinates and isotropic or equivalent isotropic displacement parameters (Å^2^)

**Table:**

	*x*	*y*	*z*	*U*_iso_*/*U*_eq_	
Cu1	0.26351 (7)	0.42966 (2)	0.96727 (3)	0.03433 (17)	
F1	0.1254 (4)	0.56914 (13)	0.76852 (17)	0.0710 (8)	
F2	−0.0108 (5)	0.67144 (15)	0.6908 (2)	0.0943 (11)	
F3	−0.0237 (5)	0.76847 (14)	0.7904 (3)	0.0997 (12)	
F4	0.1064 (5)	0.76414 (13)	0.9711 (2)	0.0947 (11)	
F5	−0.2468 (5)	0.37774 (16)	0.6457 (2)	0.0933 (11)	
F6	−0.2536 (6)	0.39045 (19)	0.4738 (2)	0.1363 (17)	
F7	0.0539 (8)	0.43382 (19)	0.4210 (2)	0.1446 (19)	
F8	0.3701 (7)	0.4633 (2)	0.5452 (3)	0.1405 (17)	
N1	0.2919 (5)	0.43593 (14)	1.1012 (2)	0.0332 (8)	
N2	0.3007 (5)	0.34263 (15)	0.9999 (2)	0.0369 (8)	
O1	0.1742 (4)	0.50970 (13)	0.93618 (18)	0.0414 (7)	
O2	0.4181 (4)	0.56062 (12)	1.01997 (18)	0.0405 (7)	
O3	0.2454 (4)	0.41553 (13)	0.83942 (18)	0.0444 (8)	
O4	−0.0627 (4)	0.39979 (14)	0.8205 (2)	0.0528 (8)	
O5	0.5762 (5)	0.35119 (16)	0.8084 (3)	0.0868 (13)	
H5A	0.4904	0.3742	0.8189	0.104*	
H5B	0.6795	0.3706	0.8140	0.104*	
C1	0.2693 (6)	0.55631 (18)	0.9608 (3)	0.0350 (10)	
C2	0.1864 (5)	0.61257 (18)	0.9132 (3)	0.0370 (10)	
C3	0.1176 (6)	0.6160 (2)	0.8215 (3)	0.0465 (12)	
C4	0.0502 (7)	0.6682 (2)	0.7804 (4)	0.0586 (14)	
C5	0.0448 (7)	0.7175 (2)	0.8309 (4)	0.0636 (15)	
C6	0.1115 (7)	0.7149 (2)	0.9221 (4)	0.0594 (14)	
C7	0.1842 (6)	0.6634 (2)	0.9644 (3)	0.0466 (11)	
H7	0.2315	0.6625	1.0266	0.056*	
C8	0.0790 (7)	0.40866 (18)	0.7915 (3)	0.0377 (10)	
C9	0.0673 (7)	0.41365 (19)	0.6907 (3)	0.0443 (11)	
C10	−0.0898 (8)	0.3995 (2)	0.6260 (3)	0.0610 (14)	
C11	−0.0961 (11)	0.4054 (3)	0.5346 (4)	0.080 (2)	
C12	0.0596 (13)	0.4274 (3)	0.5097 (4)	0.090 (2)	
C13	0.2181 (11)	0.4413 (3)	0.5731 (4)	0.0830 (19)	
C14	0.2248 (8)	0.4351 (2)	0.6621 (3)	0.0648 (15)	
H14	0.3356	0.4452	0.7045	0.078*	
C15	0.2727 (6)	0.48237 (19)	1.1508 (3)	0.0403 (10)	
H15	0.2219	0.5170	1.1220	0.048*	
C16	0.3258 (6)	0.4814 (2)	1.2446 (3)	0.0492 (12)	
H16	0.3102	0.5149	1.2774	0.059*	
C17	0.4000 (7)	0.4316 (2)	1.2880 (3)	0.0522 (12)	
H17	0.4380	0.4310	1.3507	0.063*	
C18	0.4194 (6)	0.3809 (2)	1.2385 (3)	0.0427 (11)	
C19	0.3594 (5)	0.38506 (18)	1.1446 (3)	0.0341 (10)	
C20	0.3611 (6)	0.33451 (18)	1.0892 (3)	0.0360 (10)	
C21	0.4190 (6)	0.2798 (2)	1.1298 (3)	0.0471 (12)	
C22	0.4042 (7)	0.2311 (2)	1.0711 (4)	0.0628 (15)	
H22	0.4401	0.1936	1.0938	0.075*	
C23	0.3376 (8)	0.2392 (2)	0.9815 (4)	0.0663 (15)	
H23	0.3239	0.2069	0.9427	0.080*	
C24	0.2888 (7)	0.2959 (2)	0.9466 (3)	0.0523 (13)	
H24	0.2472	0.3009	0.8845	0.063*	
C25	0.4879 (7)	0.3249 (2)	1.2765 (3)	0.0589 (14)	
H25	0.5346	0.3220	1.3386	0.071*	
C26	0.4871 (7)	0.2767 (2)	1.2254 (3)	0.0592 (14)	
H26	0.5312	0.2409	1.2525	0.071*	

### Atomic displacement parameters (Å^2^)

**Table:**

	*U*^11^	*U*^22^	*U*^33^	*U*^12^	*U*^13^	*U*^23^
Cu1	0.0356 (3)	0.0364 (3)	0.0293 (3)	−0.0019 (2)	0.0041 (2)	0.0010 (2)
F1	0.095 (2)	0.063 (2)	0.0478 (16)	0.0002 (17)	0.0018 (15)	0.0055 (15)
F2	0.113 (3)	0.091 (3)	0.066 (2)	0.003 (2)	−0.0062 (19)	0.0388 (18)
F3	0.095 (3)	0.055 (2)	0.140 (3)	0.0138 (18)	0.009 (2)	0.045 (2)
F4	0.114 (3)	0.048 (2)	0.126 (3)	0.0141 (19)	0.038 (2)	−0.0052 (19)
F5	0.071 (2)	0.116 (3)	0.077 (2)	−0.016 (2)	−0.0150 (18)	−0.025 (2)
F6	0.158 (4)	0.136 (4)	0.070 (2)	0.022 (3)	−0.063 (2)	−0.034 (2)
F7	0.255 (6)	0.141 (4)	0.0347 (18)	0.034 (4)	0.027 (3)	0.005 (2)
F8	0.178 (4)	0.178 (4)	0.087 (3)	−0.033 (4)	0.073 (3)	0.023 (3)
N1	0.037 (2)	0.031 (2)	0.0311 (17)	0.0036 (16)	0.0072 (15)	0.0034 (15)
N2	0.034 (2)	0.036 (2)	0.041 (2)	−0.0038 (16)	0.0092 (17)	−0.0027 (16)
O1	0.0372 (17)	0.0368 (18)	0.0440 (17)	−0.0052 (14)	−0.0033 (14)	0.0069 (14)
O2	0.0276 (16)	0.0471 (19)	0.0430 (16)	−0.0006 (14)	0.0003 (14)	0.0011 (13)
O3	0.0356 (18)	0.063 (2)	0.0315 (15)	−0.0012 (15)	0.0023 (14)	−0.0011 (14)
O4	0.042 (2)	0.063 (2)	0.053 (2)	−0.0077 (17)	0.0102 (16)	−0.0065 (16)
O5	0.061 (2)	0.072 (3)	0.137 (4)	−0.018 (2)	0.042 (2)	−0.044 (2)
C1	0.028 (2)	0.042 (3)	0.037 (2)	0.004 (2)	0.0105 (19)	0.004 (2)
C2	0.025 (2)	0.037 (3)	0.048 (3)	−0.0019 (19)	0.005 (2)	0.007 (2)
C3	0.041 (3)	0.045 (3)	0.052 (3)	−0.006 (2)	0.007 (2)	0.008 (2)
C4	0.048 (3)	0.060 (4)	0.064 (3)	−0.002 (3)	0.005 (3)	0.027 (3)
C5	0.049 (3)	0.048 (3)	0.093 (4)	0.007 (3)	0.014 (3)	0.029 (3)
C6	0.050 (3)	0.035 (3)	0.097 (4)	0.002 (2)	0.023 (3)	−0.001 (3)
C7	0.040 (3)	0.042 (3)	0.058 (3)	0.000 (2)	0.012 (2)	0.002 (2)
C8	0.041 (3)	0.033 (2)	0.037 (2)	0.003 (2)	0.004 (2)	−0.0046 (19)
C9	0.053 (3)	0.040 (3)	0.036 (2)	0.006 (2)	0.003 (2)	−0.006 (2)
C10	0.064 (4)	0.052 (3)	0.057 (3)	0.002 (3)	−0.007 (3)	−0.013 (3)
C11	0.110 (6)	0.066 (4)	0.042 (3)	0.020 (4)	−0.031 (4)	−0.014 (3)
C12	0.154 (7)	0.072 (5)	0.035 (3)	0.024 (5)	0.006 (4)	−0.001 (3)
C13	0.116 (6)	0.079 (5)	0.059 (4)	0.000 (4)	0.031 (4)	0.011 (3)
C14	0.087 (4)	0.071 (4)	0.037 (3)	−0.002 (3)	0.016 (3)	0.009 (3)
C15	0.039 (3)	0.038 (3)	0.046 (3)	0.002 (2)	0.014 (2)	0.003 (2)
C16	0.054 (3)	0.059 (3)	0.037 (3)	−0.001 (3)	0.015 (2)	−0.011 (2)
C17	0.051 (3)	0.074 (4)	0.032 (2)	−0.005 (3)	0.009 (2)	0.003 (3)
C18	0.037 (3)	0.049 (3)	0.041 (3)	−0.003 (2)	0.008 (2)	0.010 (2)
C19	0.027 (2)	0.041 (3)	0.034 (2)	−0.0019 (19)	0.0068 (18)	0.0064 (19)
C20	0.028 (2)	0.038 (3)	0.043 (3)	−0.0009 (19)	0.0113 (19)	0.007 (2)
C21	0.041 (3)	0.037 (3)	0.066 (3)	0.002 (2)	0.018 (2)	0.011 (2)
C22	0.063 (4)	0.035 (3)	0.093 (4)	0.003 (3)	0.024 (3)	0.011 (3)
C23	0.078 (4)	0.039 (3)	0.088 (4)	−0.007 (3)	0.031 (3)	−0.013 (3)
C24	0.063 (3)	0.045 (3)	0.051 (3)	−0.010 (2)	0.016 (3)	−0.008 (2)
C25	0.058 (3)	0.067 (4)	0.050 (3)	0.003 (3)	0.008 (3)	0.024 (3)
C26	0.060 (3)	0.050 (3)	0.067 (4)	0.010 (3)	0.015 (3)	0.033 (3)

### Geometric parameters (Å, º)

**Table:**

Cu1—O1	1.942 (3)	C6—C7	1.374 (6)
Cu1—O3	1.948 (3)	C7—H7	0.9300
Cu1—N1	2.008 (3)	C8—C9	1.523 (6)
Cu1—N2	2.032 (3)	C9—C10	1.353 (6)
Cu1—O2^i^	2.262 (3)	C9—C14	1.392 (7)
F1—C3	1.341 (5)	C10—C11	1.389 (8)
F2—C4	1.336 (6)	C11—C12	1.357 (9)
F3—C5	1.346 (5)	C12—C13	1.348 (9)
F4—C6	1.345 (6)	C13—C14	1.352 (7)
F5—C10	1.327 (6)	C14—H14	0.9300
F6—C11	1.329 (6)	C15—C16	1.391 (6)
F7—C12	1.349 (6)	C15—H15	0.9300
F8—C13	1.356 (7)	C16—C17	1.351 (6)
N1—C15	1.319 (5)	C16—H16	0.9300
N1—C19	1.359 (5)	C17—C18	1.396 (6)
N2—C24	1.323 (5)	C17—H17	0.9300
N2—C20	1.340 (5)	C18—C19	1.398 (5)
O1—C1	1.265 (5)	C18—C25	1.430 (6)
O2—C1	1.232 (5)	C19—C20	1.423 (6)
O2—Cu1^i^	2.262 (3)	C20—C21	1.402 (6)
O3—C8	1.257 (5)	C21—C22	1.407 (6)
O4—C8	1.218 (5)	C21—C26	1.427 (6)
O5—H5A	0.8500	C22—C23	1.350 (7)
O5—H5B	0.8499	C22—H22	0.9300
C1—C2	1.516 (5)	C23—C24	1.402 (7)
C2—C3	1.371 (6)	C23—H23	0.9300
C2—C7	1.391 (6)	C24—H24	0.9300
C3—C4	1.369 (6)	C25—C26	1.339 (7)
C4—C5	1.361 (7)	C25—H25	0.9300
C5—C6	1.362 (7)	C26—H26	0.9300
			
O1—Cu1—O3	88.14 (12)	C9—C10—C11	122.3 (6)
O1—Cu1—N1	97.58 (13)	F6—C11—C12	121.5 (6)
O3—Cu1—N1	174.29 (13)	F6—C11—C10	119.8 (7)
O1—Cu1—N2	168.38 (13)	C12—C11—C10	118.6 (6)
O3—Cu1—N2	93.47 (13)	C13—C12—F7	121.1 (8)
N1—Cu1—N2	80.97 (13)	C13—C12—C11	119.9 (6)
O1—Cu1—O2^i^	101.59 (11)	F7—C12—C11	119.0 (7)
O3—Cu1—O2^i^	86.16 (11)	C12—C13—C14	121.5 (7)
N1—Cu1—O2^i^	92.52 (12)	C12—C13—F8	117.9 (6)
N2—Cu1—O2^i^	90.01 (12)	C14—C13—F8	120.6 (6)
C15—N1—C19	117.9 (3)	C13—C14—C9	120.5 (6)
C15—N1—Cu1	129.7 (3)	C13—C14—H14	119.7
C19—N1—Cu1	112.0 (3)	C9—C14—H14	119.7
C24—N2—C20	118.2 (4)	N1—C15—C16	122.3 (4)
C24—N2—Cu1	129.5 (3)	N1—C15—H15	118.8
C20—N2—Cu1	112.0 (3)	C16—C15—H15	118.8
C1—O1—Cu1	125.4 (3)	C17—C16—C15	119.9 (4)
C1—O2—Cu1^i^	139.4 (3)	C17—C16—H16	120.0
C8—O3—Cu1	115.7 (3)	C15—C16—H16	120.0
H5A—O5—H5B	108.7	C16—C17—C18	119.8 (4)
O2—C1—O1	126.9 (4)	C16—C17—H17	120.1
O2—C1—C2	117.5 (4)	C18—C17—H17	120.1
O1—C1—C2	115.6 (4)	C17—C18—C19	116.8 (4)
C3—C2—C7	118.4 (4)	C17—C18—C25	125.2 (4)
C3—C2—C1	122.9 (4)	C19—C18—C25	118.0 (4)
C7—C2—C1	118.7 (4)	N1—C19—C18	123.0 (4)
F1—C3—C4	117.7 (4)	N1—C19—C20	116.3 (3)
F1—C3—C2	120.8 (4)	C18—C19—C20	120.7 (4)
C4—C3—C2	121.3 (5)	N2—C20—C21	124.1 (4)
F2—C4—C5	119.1 (5)	N2—C20—C19	116.5 (4)
F2—C4—C3	120.8 (5)	C21—C20—C19	119.3 (4)
C5—C4—C3	120.1 (5)	C20—C21—C22	116.0 (4)
F3—C5—C4	119.9 (5)	C20—C21—C26	119.3 (4)
F3—C5—C6	120.6 (5)	C22—C21—C26	124.6 (5)
C4—C5—C6	119.5 (5)	C23—C22—C21	119.6 (5)
F4—C6—C5	118.9 (5)	C23—C22—H22	120.2
F4—C6—C7	119.9 (5)	C21—C22—H22	120.2
C5—C6—C7	121.2 (5)	C22—C23—C24	120.3 (5)
C6—C7—C2	119.4 (5)	C22—C23—H23	119.8
C6—C7—H7	120.3	C24—C23—H23	119.8
C2—C7—H7	120.3	N2—C24—C23	121.6 (5)
O4—C8—O3	124.9 (4)	N2—C24—H24	119.2
O4—C8—C9	121.6 (4)	C23—C24—H24	119.2
O3—C8—C9	113.5 (4)	C26—C25—C18	122.1 (5)
C10—C9—C14	117.1 (5)	C26—C25—H25	118.9
C10—C9—C8	123.9 (5)	C18—C25—H25	118.9
C14—C9—C8	118.9 (4)	C25—C26—C21	120.5 (4)
F5—C10—C9	122.2 (5)	C25—C26—H26	119.8
F5—C10—C11	115.5 (5)	C21—C26—H26	119.8

Symmetry code: (i) −*x*+1, −*y*+1, −*z*+2.

### Hydrogen-bond geometry (Å, º)

**Table:**

*D*—H···*A*	*D*—H	H···*A*	*D*···*A*	*D*—H···*A*
O5—H5*A*···O3	0.85	2.08	2.918 (5)	168
O5—H5*B*···O4^ii^	0.85	1.95	2.785 (5)	168

Symmetry code: (ii) *x*+1, *y*, *z*.
