# Supplementary figures and images for: Crystal structure of bis­(μ-2,3,4,5-tetra­fluoro­benzoato-κ2 O:O′)bis­[(1,10-phen­anthroline-κ2 N:N′)(2,3,4,5-tetra­fluoro­benzoato-κO)copper(II)] dihydrate
Source: Acta Crystallogr Sect E Struct Rep Online. 2014 Oct 11;70(Pt 11):m365–6. doi: 10.1107/S1600536814022065 (PMC4257334; doi:10.1107/S1600536814022065)

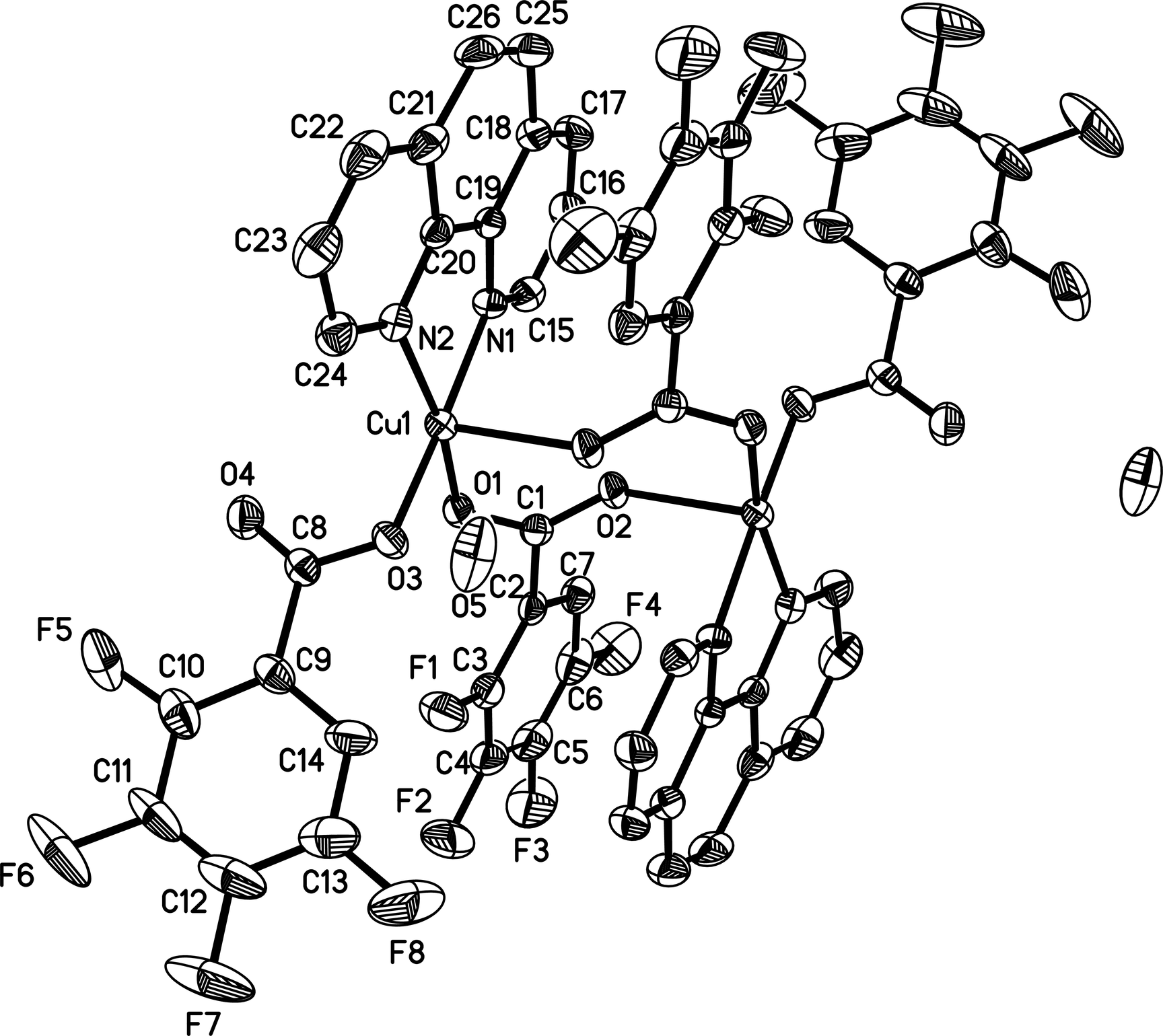

Supplement: Supplementary file 3 [file e-70-0m365-fig1.tif]

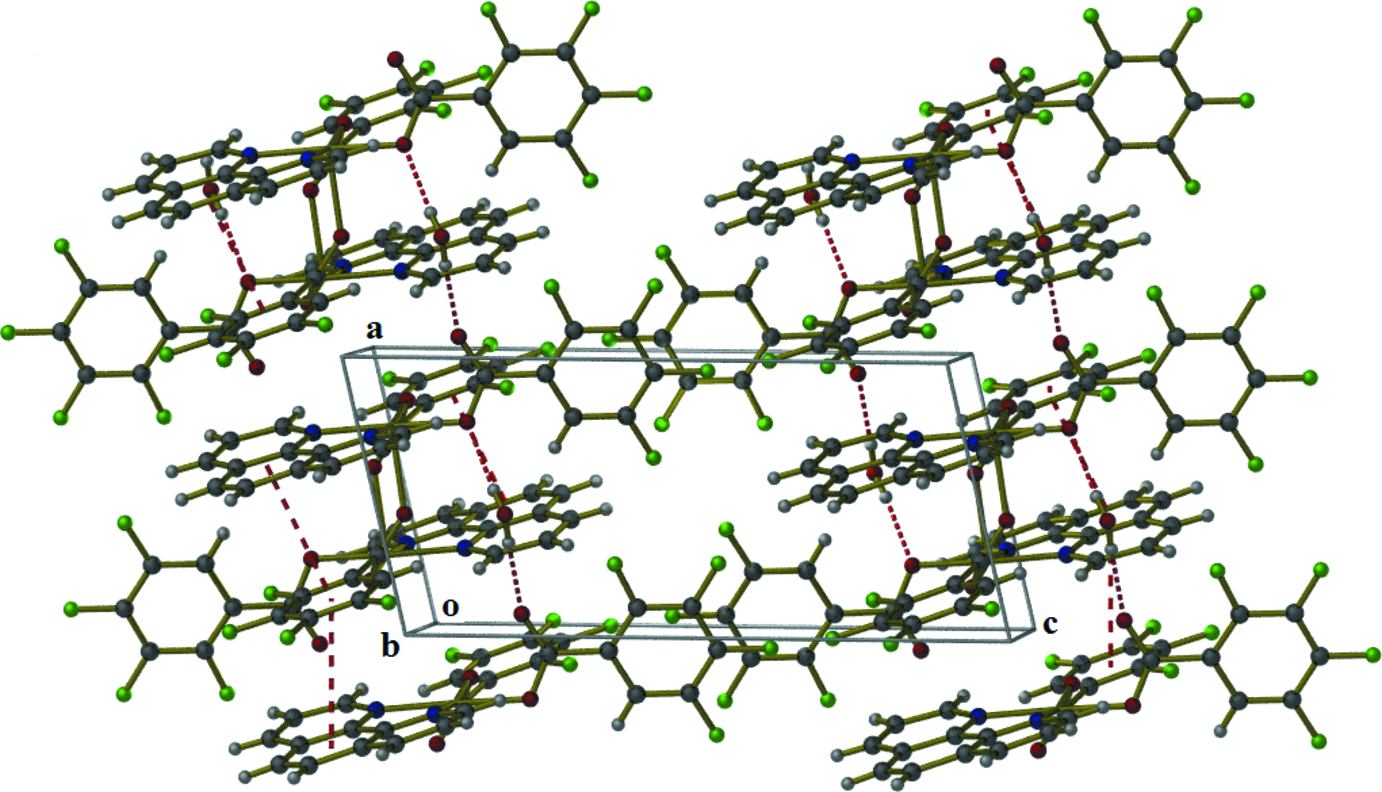

Supplement: Supplementary file 4 [file e-70-0m365-fig2.tif]
